# Does the Domestication Syndrome Apply to the Domestic Pig? Not Completely

**DOI:** 10.3390/ani12182458

**Published:** 2022-09-17

**Authors:** Edoardo Collarini, Marika Gioia, Giada Cordoni, Ivan Norscia

**Affiliations:** Department of Life Sciences and Systems Biology, University of Torino (DBIOS), Via Accademia Albertina 13, 20123 Torino, Italy

**Keywords:** *Sus scrofa*, swine, domestication, artificial selection, Neolithic, new stone age, play, aggression, *Homo sapiens*, livestock

## Abstract

**Simple Summary:**

An interesting anthropological question is whether, over the last 10,000 years, humans have domesticated animals by actually selecting the tamest and least aggressive individuals. These characteristics—known as ‘domestication syndrome’—should be present from the very early stages of life of domesticated animals. Because social play is the most important ‘friendly’ contact between very young individuals, we verified whether piglets (with both domestic pig parents) were more playful and less aggressive than hybrid pups (with wild boar father and domestic pig mother). To this purpose, we investigated three litters of piglets and three litters of wild boar hybrids, all raised in northern Italy (Parva Domus farm, Cavagnolo, Torino) in the same grassland/woodland environment and under similar farming conditions. We found that piglets played more and in a ‘less chaotic’ way than wild boar hybrids, especially after the first three weeks of life but also that piglets were more aggressive than hybrids, with piglet aggression being more frequently unbalanced in favour of one individual. Thus, the domestication syndrome does not fully apply to either social play or aggression, possibly because artificial selection may have produced greater tameness of pigs towards humans than towards other pigs.

**Abstract:**

The ‘domestication syndrome’ defines a suite of features that domesticated animals possess as the result of the artificial selection operated by *Homo sapiens* since the Neolithic. An interesting anthropological question is whether such features, including increased tameness and reduced aggression, apply to all domesticated forms. We investigated this issue in the domestic pig (*Sus scrofa*). We video-recorded and analysed aggression and social play (mostly play-fighting) sessions from piglets (three litters; n = 24) and wild boar hybrids (domestic pig mother x wild boar father; three litters; n = 27) from 6–50 days of age, raised in the same woodland/grassland habitat and extensive farming management (ethical farm ‘Parva Domus’, Cavagnolo, Torino). Play and aggression session structure was assessed via Asymmetry (AI; offensive/defensive pattern balance), Shannon (H′; pattern variability), and Pielou (J; pattern evenness) indices. We found that piglets played more (especially after the 20th day of life) and engaged in less variable and uniform sessions than wild boar hybrids. Compared to hybrids, piglets showed less variable but more frequent (especially when approaching weaning) and asymmetrical aggressive events. Thus, the domestication syndrome does not seem to fully apply to either social play or aggression, possibly because artificial selection has produced greater tameness of pigs towards humans than towards conspecifics.

## 1. Introduction

Over the past 11,000 years humans have domesticated a wide range of animal species as livestock and household pets [1]. Domestication is an evolutionary process through which animals are artificially selected and become adapted to humans and to the captive environment [2,3]. Via this process, humans modulate different aspects of the natural history of the domesticated species, including distribution, breeding, diet, and behaviour [4,5,6]. From a behavioural point of view, the most selected characteristics undergoing domestication are low levels of aggression and increased tameness, which correspond to neotenous features [5,7,8,9,10,11]. For example, dogs are neotenous compared to their wild counterparts-the wolves [12,13]—because artificial selection has favoured individuals showing more playful and less aggressive behaviours [14,15,16]. Such a suite of behavioural traits—often accompanied by physio-morphological features (e.g., testosterone levels, cranial morphology)—is known as ‘domestication syndrome’ [17,18]. As a result, when compared with their wild counterparts, domesticated animals commonly express increased levels of prosocial behaviours associated with positive emotions, such as sociability and playfulness, and lowered levels of reactive behaviours associated with negative emotions, such as stress, fear, and overt conflict [19,20,21,22,23].

As concerns household pets, increases in tameness seem to have characterised the domestication of the cat (*Felis catus*), starting from wild species with more docile temperaments (probably *Felis libyca*) [24]. The behavioural modification from fear–aggressiveness to tameness is also a trait that has probably marked the initial domestication of dogs (*Canis lupus familiaris*), leading to increased playfulness and decreased persistent aggression in modern breeds (although not in a consistent way across time and breeds) [19,25]. Moreover, compared to wild cavies (*Cavia aperea*), domestic guinea pigs (*Cavia porcellus*) are characterised by higher levels of social activity and lower levels of exploration, risk-taking, and cortisol reactivity [18].

As concerns livestock, the increased docility and suppression of the fight-or-flight response seem to be traits that have been selected during the domestication of goats (*Capra hircus*) and sheep (*Ovis aries*) (compared to wild ancestors, such as the Persian wild goat *Capra aegagrus* or West Asiatic mouflon *Ovis orientalis*, respectively) [4,26]. Despite these examples, the literature reports very few case studies that directly compare the behaviour of domestic species with the behaviour of their wild counterparts in order to quantitatively assess—from an anthropological perspective—the behavioural differences possibly associated with the domestication syndrome. To fill in part this gap, we carried out a direct behavioural comparison between wild boar hybrids and domestic pigs (*Sus scrofa*). The domestic pig is an excellent model to investigate because its initial domestication (which occurred independently in at least two regions, Northern Mesopotamia and China) dates to around 10,000 years ago [27,28,29]. Domestic pigs show sociality and gregariousness, low reactions to humans, and low fear reactions [1,30,31]. Recently, Price and Hongo [27] encouraged the adoption of a multimethod approach to shed light on the pig domestication process, which has led to genotypical/phenotypical adaptations to human management. We posit that the comparison between domestic and wild forms could be part of such a multimethod approach. As a matter of fact, although the behavioural repertoire of pigs is as rich as the repertoire of their wild counterpart (the wild boar) [31,32,33], it is possible to find differences caused by changes in sensitivity to the stimuli and there may be quantitative variations in the level of expression of certain behavioural patterns [31,33,34,35,36]. For example, wild boar and feral pigs (pigs that have lived in wild conditions for generations) are more reactive than domestic pigs to the presence of predators, including humans [37]. In this regard, the process of domestication probably produced changes in behavioural traits of domestic pigs [31,38,39,40,41]. Although one important selection criterion by early breeders was the fast growth rate [42], aggression levels seem to have played a role during the early stages of domestication and the genetic causes (linked to variations in the serotonin pathways) are likely shared across domestic breeds [43]. Moreover, social traits that emerged in ancestral suids millions of years ago, have been altered by human selection in the Holocene during the domestication process, to favour docility [27].

In this study, we compared the aggressive and social play behaviour in three litters of piglets and three litters of wild boar x domestic pig hybrids (hereafter wild boar hybrids) raised under the same natural (woodland/grassland) and management conditions. The immature phase is particularly suitable because, although a certain degree of sociality is maintained in adults, especially in females, sociality tends to decrease with aging. As a matter of fact, social contacts including play, gradually decrease after the first phases of life [39,44,45]. The immature phase also reduces possible biases related to aspects of social life that may be learned from experience. To investigate what behavioural traits of pigs may have been moulded by the domestication process (which foresees increased docility and decreased aggression), we formulated the following predictions.

### 1.1. Prediction 1

During the early days of life, play behaviour, including play-fighting (or rough-and-tumble play), is the main social contact that occurs between piglets [36,41,46,47]. Play-fighting helps piglets to develop the motor and social skills that are necessary to face opponents, enhance conflict resolution, individual recognition, group cohesion, and social bonds [44,46,48]. Following the domestication syndrome, domesticated animals are expected to show higher levels of affiliation and playfulness compared to their wild counterparts [19,21]. Hence, we posited that play-fighting would be more expressed in piglets than in wild boar hybrid pups (*Prediction 1a*). 

Successful playful interactions are characterized by long duration and high balance because subjects are more skilled in managing the high pattern variability typical of play [49,50,51,52,53]. Thus, if the domestication syndrome applies to the domestic pigs and domestic pigs are more ‘skilled’ players than their wild counterparts, we expected that piglets would be more capable to balance and prolong highly variable play fight sessions (*Prediction 1b*). 

Social play is a widespread behaviour that is mostly typical of immature subjects and decreases as age increases, also in pigs [41,47]. Hence, if the domestication syndrome applies to the domestic pigs and has led to the presence of behavioural neotenous traits, we expected that piglets—compared to wild boar hybrids—would show higher levels of social play at later stages of the lactation period, when weaning approaches (*Prediction 1c*). 

### 1.2. Prediction 2 

In adult pigs, conflicts can be observed to access food and establish hierarchy [31]. In piglets, aggressive behaviours can be observed since the very first days of life when they compete for the most productive udders [41,46,54]. However, it is also possible to observe aggressive behaviour among immature subjects out of the context of lactation [45]. 

According to the domestication syndrome, domesticated animals should show lower levels of aggression compared to their wild counterparts [55,56,57]. Hence, we expected to find lower levels of aggression in piglets compared to wild boar hybrids (*Prediction 2a*). In species with low tolerance levels, individuals fiercely compete to gain advantage over their groupmates, thus establishing higher ranking positions and obtaining priority access to desirable resources [58,59,60]. In this respect, aggressive social groups should show real fights that are highly directional (unbalanced in favour of dominants) and predictable in terms of offensive/defensive patterns. Hence, if the domestic form is more ‘peaceful’ than the wild form, as predicted by the domestication syndrome, we expected that aggression in piglets would be shorter and less directional and predictable than in wild boar hybrids (*Prediction 2b*).

Aggressive events increase as age increases and sexual maturity is approached, because aggression is used to access food and to set up dominance relationships [31,41]. If the domestication syndrome applies to domestic pigs and, again, has led to the presence of behavioural neotenous traits, we expect aggressive levels to be lower in piglets than in wild boar hybrids at any developmental stage (*Prediction 2c*).

## 2. Materials and Methods

### 2.1. Ethical Statement

This research was purely observational and non-invasive; therefore, it did not require any specific permission. Piglets were not removed from their group during observations, and they were free to perform their ordinary maintenance and social activities and to stay out of our sight if wanted. 

### 2.2. The Study Group

The research was carried out on three domestic piglet litters (mixed breed: Large White x Parma Black) and three wild boar hybrid litters (Large White breed × wild boar) at the “Ethical Farm Parva Domus”, in Cavagnolo, Turin (Italy). The study was conducted from June to December 2018 and included: (i) 24 piglets (11 females, 13 males; Table 1), with different mothers and the same father (Parma Black); and (ii) 27 wild boar hybrids (13 females, 14 males; Table 1) with different mothers and same father (one wild boar captured a few days after mating with sows). All sows were housed in spaces of grassland–woodland natural habitat, and during the observation period all the sows stayed isolated with their litters either with a physical barrier (fence, for the piglet mothers) or by providing food in a separate space (≥100 m^2^). This condition was already present and part of the farmer management and not changed for the study purposes. Sows received food pellets (Ciclo Unico P, SILDAMIN) each morning between 8:30 a.m. and 10:30 a.m. Piglets and wild boar hybrids were able to opportunistically integrate maternal milk with roots, leaves, and fruits found in the natural environment or with pellets left by their mother. Piglet tails and teeth were kept intact, and males (of both hybrids and piglets) were castrated. The animals followed the natural day/night cycle and did not show any aberrant or stereotypical behaviours. To facilitate the identification, each individual had a unique marking with spray Raidex© for livestock; the marking was renewed every 4–7 days depending on weather conditions. During the video recording, the observers stayed around 10 m from piglets and sows.

### 2.3. Data Collection and Video Analyses

**Figure 1:**
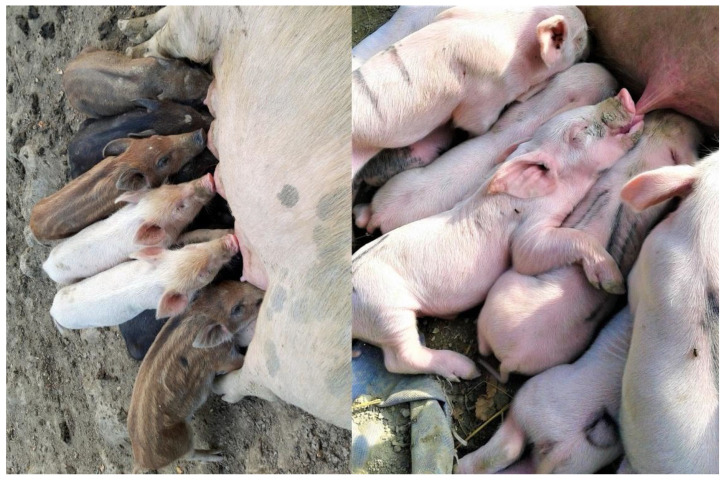
Wild boar hybrids (**left**) and piglets (**right**) during lactation (piglets are marked for individual identification).

Owing to the spatial separation of sows and their litters from others, all observed interactions occurred within litters. The behavioural patterns were video-recorded from Jun–Jul 2018 for hybrids and Sept–Dec 2018 for piglets by using HD/Full HD Sony HDR-XR200 and Panasonic HC-W3580 cameras. We collected and analysed 15.92 h (Mean ± SE: 5.31 h ± 0.60) and 12.05 h (Mean ± SE: 4.02 h ± 0.46) for piglets and wild boar hybrids, respectively. During the first week after birth, all subjects were habituated to the presence of the observers and data collection started around the end of the first week of life. Via the all occurrences sampling method [61], we recorded social play (n = 477) and aggression (n = 166) sessions in both piglets and hybrids (Figure 1).

Prior to starting the video analysis, video coders (M.G., Ed.C., M.C.) were supervised by G.C. and I.N. in behavioural coding and the video analysis started when the interobserver reliability scores measured via Cohen’s k reached 0.83. The interobserver reliability between video coders was calculated using the R function ‘cohen.cappa’ and libraries ‘irr’ and ‘psych’ (R version 3.5.3). The videos were analysed frame by frame or slow motion via freeware software VLC 2.2.1 (jump-to-time extension). For each interaction session, we extracted from videos the following information: (1) the identity of the subjects involved, (2) individual features (sex, age), (3) behavioural patterns performed (Table 2), (4) time of each pattern and the length of the session (s). Finally, we defined three periods spanning 14 days of piglet life from the 6th to the 50th day of life: T_1_ (6–20 days); T_2_ (21–35 days); and T_3_ (36–50 days).

### 2.4. Operational Definitions and Structural Indices

We considered a play session as started when a piglet directed any playful pattern (see Table 2) towards the littermate and finished when both players stopped the interaction, with one of them moving away or with a third subject interrupting the session. Play sessions were considered as different if the play interaction stopped for at least 10 s [41].

We considered an aggression to have started when a piglet directed any aggressive pattern (see Table 3) towards a litter-mate and it ended with one of the opponents moving or fleeing away. Two aggressive sessions were considered as different if the real fight interaction stopped for at least 10 s [41]. The duration and frequency of social play and aggressive events were calculated considering all sessions. 

For the purpose of this study, play fights and real fights included at least two behavioural patterns listed in the ethogram [44,46,47,62,63,64,65], Table 2, and Table 3; examples of play fight sessions—Videos S1 and S2; and examples of real fight sessions—Videos S3 and S4). For both play and real fights, the patterns were classified as *Offensive* (O, unidirectional patterns performed to threat, attack, pursue, and injure the opponent), *Defensive/Submissive* (D/S, patterns of body protection, contact avoidance, and submission), and Neutral (N, neither offensive nor defensive; play fight—Table 2; real fight—Table 3). For each play fight and real fight session, we calculated different indices in order to evaluate the distribution and variability of the motor patterns performed within the session and the symmetry of the interaction. In particular, we considered the indices listed below.

#### Asymmetry Index (AI)

The play Asymmetry Index (pAI) [41,66,67] was used to quantify the level of play-fighting asymmetry. For each interaction we calculated pAI as follows: ‘the number of offensive patterns by A towards B *plus* the number of defensive patterns by B towards A’ *minus* ‘the number of offensive patterns by B towards A *plus* the number of defensive patterns by A towards B’ divided by ‘the total number of patterns performed by both playmates’. The formula of pAI is reported below:

$$PAI=\frac{\left(offensive_{A\to B}+defensive_{B\to A}\right)-\left(offensive_{B\to A}+defensive_{A\to B}\right)}{\left(offensive_{A\to B}+defensive_{B\to A}\right)+\left(offensive_{B\to A}+defensive_{A\to B}\right)+neutral_{A+B}}$$

Based on pAI, we also calculated the aggression Asymmetry Index (aAI) to quantify the level of real fight asymmetry. For each interaction we calculated aAI as follows: ‘the number of offensive patterns by A towards B *plus* the number of defensive patterns by B towards A’ *minus* ‘the number of offensive patterns by B towards A *plus* the number of defensive patterns by A towards B’ divided by ‘the total number of patterns performed by both opponents’. The formula of aAI is reported below:

$$AAI=\frac{\left(offensive_{A\to B}+defensive_{B\to A}\right)-\left(offensive_{B\to A}+defensive_{A\to B}\right)}{\left(offensive_{A\to B}+defensive_{B\to A}\right)+\left(offensive_{B\to A}+defensive_{A\to B}\right)+neutral_{A+B}}$$

Both pAI and aAI range from −1 to +1 with main values indicating (i) a complete symmetry of the session (zero), (ii) a complete asymmetry of the session in favour of A (+1), and (iii) a complete asymmetry of the session in favour of B (−1). 

The indices listed below were adapted from indices employed for measuring biodiversity in ecological studies [68,69,70]. In particular, we considered each play/real fight session as an ‘ecosystem’, including individuals (i.e., in our case all the behavioural patterns included in the session) belonging to different species (i.e., in our case the different types of behavioural patterns included in the session). Based on this approach, we calculated the Shannon and the Pielou indices. 

Shannon index: (H′; also known as Shannon’s diversity index, Shannon–Wiener index, Shannon–Weaver index, and Shannon entropy) is the most common diversity index used in ecological studies and it allows the description of both richness and evenness of a specific ecosystem [71,72,73]. Evenness refers the relative abundance of species, quantifying the equality of a community, and its value is higher if each species has equal distribution in an ecosystem [74,75,76]. The mathematical formula of the Shannon index is:

H′ = −Σ[(*n_i_/N*) * (*ln n_i_*/*N*)]

In particular, *n_i_* is the number of individuals belonging to the species *i* and *N* is the total number of individuals in a specific ecosystem. H′ values are generally between zero and five; when they are equal or higher than four indicate a great level of biodiversity and a balanced ecosystem structure. In our study, *n_i_* is represented by numbers of patterns belonging to the type *I*, and *N* is represented by the total number of patterns composing a session. A high value of H′ indicates a great behavioural pattern variability in terms of different types of patterns performed in a single session.

Pielou index (J; also known as Species evenness) derives from Shannon index and is the measure of the distribution of individuals among species within a specific ecosystem [77]. The mathematical formula of *Pielou index* is:

J = H′/H′_max_

H′ is the observed value of Shannon index, and H′_max_ is the ln*S* with *S* representing the total number of species. The values of J vary between zero and one; when they are close to one it means that individuals are even distributed among species [77]. In the present study *S* is represented by the total number of different types of behavioural patterns. To calculate the indices and run analyses on them we selected the dyads with at least two sessions of either play or real fighting.

### 2.5. Statistical Elaboration

Owing to non-normal data distribution (Kolmogorov–Smirnov, 24 ≤ n ≤ 27, 0.000 < *p* ≤ 0.045) we applied non-parametric tests at the individual level. In particular we applied the Mann–Whitney test for two independent samples [78] to compare the mean of social play and aggression frequency and duration between piglets and wild boar–pig hybrids

We applied the Friedman’s test for k dependent samples to compare social play and aggression session levels across periods. We applied the Dunn post hoc test for pairwise comparisons, with the significance level of probability adjusted downward using the Bonferroni correction.

Owing to normal data distribution (Kolmogorov–Smirnov, 10 ≤ n ≤ 58; 0.051 ≤ *p* ≤ 0.200) at the dyadic level, we applied the parametric t-test to independent samples in order to compare pAI/aAI values and H′ and J values between piglets and wild boar hybrids for either play fight and real fight sessions. Due to possible data pseudoreplication (same individuals included in different dyads), we applied a resampling procedure (bootstrapping, 10,000 permutations). All tests were carried out via SPSS 20.0 (Data_S1). The significance level was set at *p* < 0.05. A trend of significance was discussed for 0.05 ≤ *p* < 0.1.

## 3. Results

### 3.1. Social Play Levels and Structure in Piglets and Wild Boar Hybrids (Prediction 1)

**Figure 2:**
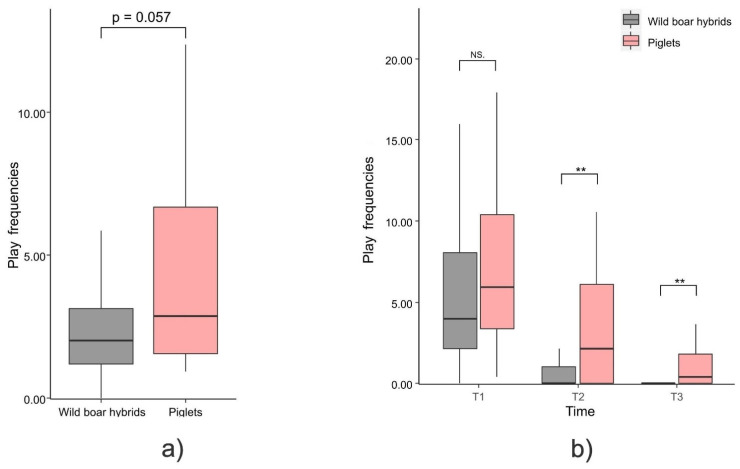
Box plot showing that: (**a**) the overallsocial play frequency tends to be higher in piglets than wild boar hybrids for the whole period (Mann–Whitney exact test; *p* = 0.057); (**b**) social play hourly frequencies (age periods: T_1_ = 6–20 days, T_2_ = 21–35 days, T_3_ = 36–50 days) are higher in piglets in T_2_ (Mann–Whitney exact test; *p* = 0.006) and in T_3_ (Mann–Whitney exact test; *p* = 0.001). There was a significant decrease in the social play levels across periods for both hybrids and piglets (Friedman’s tests, *p* < 0.05) and particularly between T_1_ and T_2_/T_3_ (Dunn’s tests: *p* < 0.05). Horizontal line: median value; box: interquartile range; vertical line: minimum and maximum values in the data. NS = non-significant, ** = *p* < 0.01.

We found a trend of significance in the comparison between the mean frequency of social play sessions between piglets and wild boar hybrids (Mann–Whitney exact test: U = 601.00, N_pigs_ = 24, N_hybrids_ = 27, *p* = 0.057; Figure 2a). 

*Temporal distribution of social play*. There was a significant decrease of the social play levels across periods (Figure 2b) and particularly from T_1_ to T_2_/T_3_ (but not between T_2_ and T_3_) for both wild boar hybrids (Friedman test: N_hybrids_ = 27, χ^2^ = 34.449, df = 2, *p* < 0.001; and Dunn test, T_2_ vs. T_1_: Q = 3.878, *p* < 0.001; T_3_ vs. T_1_: Q = 5.103, *p* < 0.001; T_3_ vs. T_2_: Q = 1.225, *p* = 0.662) and piglets (Friedman test: N_pigs_ = 24, χ^2^ = 15.404, df = 2, p < 0.001; and Dunn test, T_2_ vs. T_1_: Q = 2.742, *p* = 0.018; T_3_ vs. T_1_: Q = 3.753, *p* = 0.001; T_3_ vs. T_2_: Q = 1.010, *p* = 0.937). The comparison between piglets and wild boar hybrids within each development period (T_1_, T_2_, T_3_) revealed that piglets showed higher frequencies of social play sessions than wild boar hybrids in T_2_ (Mann–Whitney exact test: U = 563.00, N_pigs_ = 24, N_hybrids_ = 27, *p* = 0.006; Figure 2) and in T_3_ (Mann–Whitney exact test: U = 549.00, N_pigs_ = 24, N_hybrids_ = 27, *p* = 0.001; Figure 2), whereas no difference was detected in T_1_ (Mann–Whitney exact test: U = 647.00, N_pigs_ = 24, N_hybrids_ = 27, *p* = 0.299; Figure 2b).

**Figure 3:**
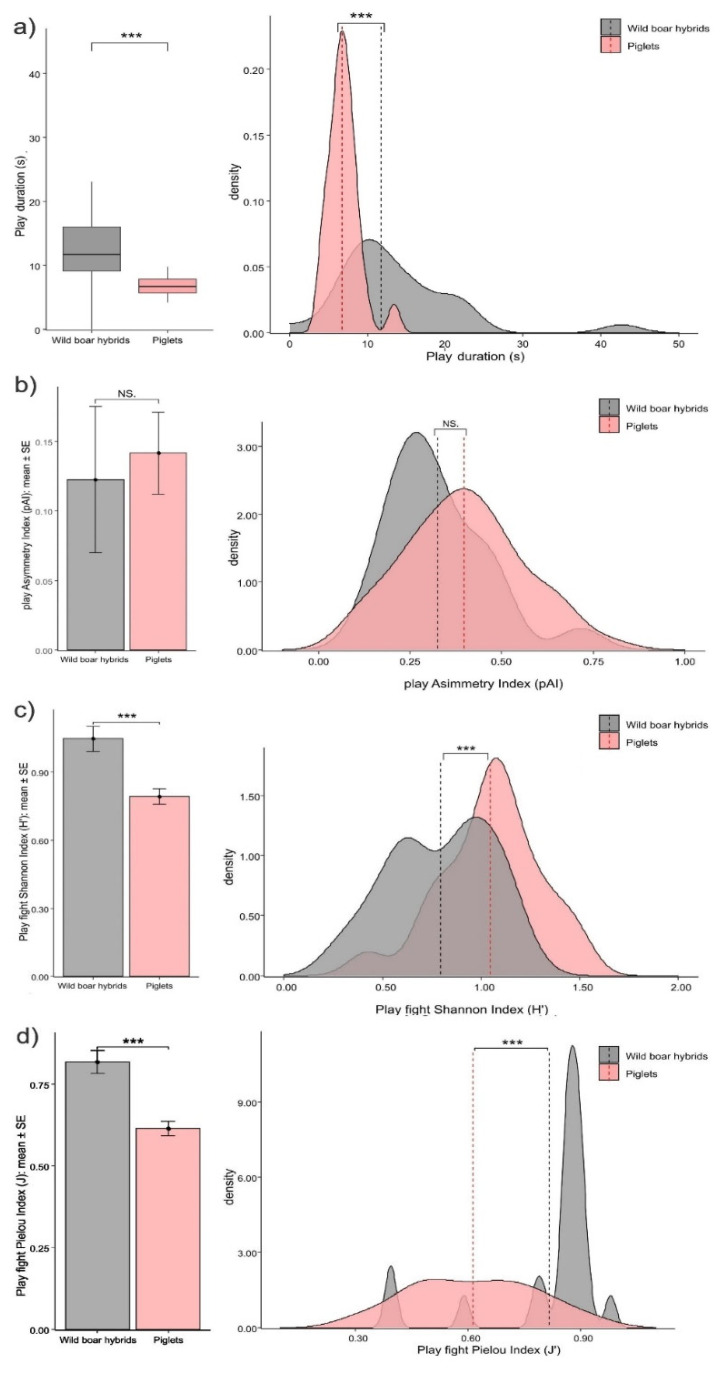
Differences in the duration of the play sessions between piglets and wild boar hybrids shown as bar plot (left) and density plot (right). (**a**) Piglets show shorter play sessions (Mann–Whitney exact test; *p* < 0.001). Density plot: vertical, dashed lines indicate median value. Bar plot: horizontal line: median value; box: interquartile range, vertical line: minimum and maximum values in the data. Differences in play fighting between piglets and wild boar hybrids are shown as error bar plot (left) and density plot (right). (**b**) Asymmetry Index (pAI) values; piglets and wild boar hybrids show comparable levels of asymmetry (*t*-test for independent samples; *p* = 0.753). (**c**) Shannon Index (H′) values; piglets show the lowest levels of play fight variability (*t*-test for independent samples; *p* < 0.001). (**d**) Pielou Index (J) values; piglets show the lowest levels of evenness in play fight (evenness) (*t*-test for independent samples; *p* < 0.001). Density plot: vertical, dashed lines indicate mean value. Density plot: vertical, dashed lines indicate mean value. Error bar plot: vertical bars; Standard Error (SE) around the mean (circles). NS = non-significant, *** = *p* < 0.001.

*Structure of social play*. Social play sessions were shorter in piglets than in wild boar hybrids (Mann–Whitney exact test: U = 392.00, N_pigs_ = 24, N_hybrids_ = 27, *p* < 0.001; Figure 3a). The asymmetry of play fight sessions (pAI) was comparable between piglets than wild boar hybrids (*t*-test for independent samples, N_pig_dyads_ = 58; N_hybrid_dyads_ = 21; t = −0.318; *p* = 0.753; Figure 3b). The level of pattern variability (Shannon Index, H′) and evenness (Pielou Index, J) were significantly lower in piglets than in wild boar hybrids (*t*-test for independent samples, N_pig_dyads_ = 58; N_hybrid_dyads_ = 21; H′: t = 3.899; *p* < 0.001; Figure 3c; J: t = 4.953 *p* < 0.001, Figure 3d). 

### 3.2. Aggression Level and Structure in Piglets vs. Wild Boar Hybrids (Prediction 2) 

**Figure 4:**
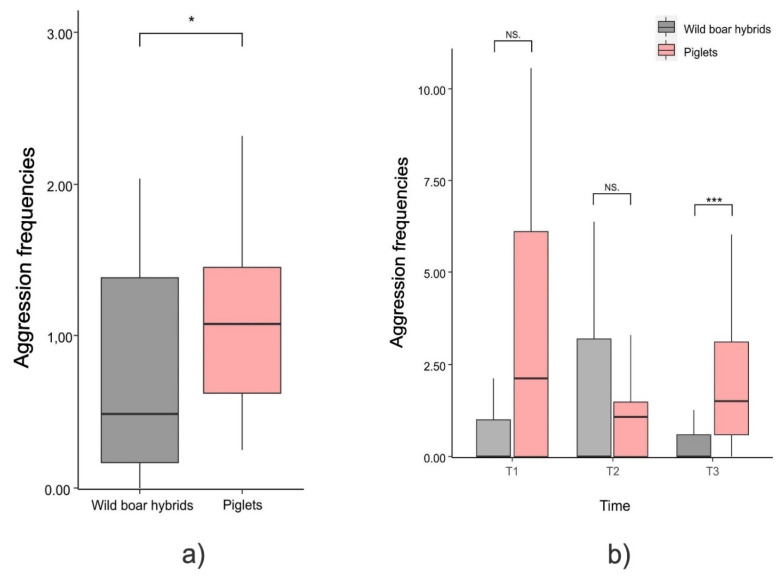
Box plot showing that: (**a**) the overall frequency of aggression are significantly higher than in piglets lower wild boar hybrids for the whole period (Mann–Whitney exact test; *p* = 0.017); (**b**) aggression hourly frequencies (age periods: T_1_ = 6–20 days, T_2_ = 21–35 days, T_3_ = 36–50 days) are higher in piglets in T_3_ (Mann–Whitney exact test; p < 0.001) but not in the other periods. No variation was observed across periods (T_1_–T_3_) in both hybrids and piglets (Friedman’s test; *p* = ns). Horizontal line: median value; box: interquartile range; vertical line: minimum and maximum values in the data. NS = non-significant, * = *p* < 0.05, *** = *p* < 0.001.

The mean frequency of aggressive sessions was significantly higher in piglets than in wild boar hybrids (Mann–Whitney exact test: U = 574.00, N_pigs_ = 24, N_hybrids_ = 27, *p* = 0.017; Figure 4a). 

*Temporal distribution of aggression*. There was no variation in the levels of aggression across development periods (T_1_–T_3_) for both wild boar hybrids (Friedman test: N_hybrids_ = 27, χ^2^ = 2.282, df = 2, *p* = 0.320) and piglets (Friedman test: N_pigs_ = 24, χ^2^ = 3.376, df = 2, *p* = 0.185). The comparison between piglets and wild boar hybrids within each development period (T_1_, T_2_, T_3_) revealed that piglets showed higher frequencies of real fight sessions in T_3_ (Mann–Whitney exact test: U = 514.50, N_pigs_ = 24, N_hybrids_ = 27, *p* < 0.001; Figure 4), whereas no difference was detected in T_1_ (Mann–Whitney exact test: U = 605.00, N_pigs_ = 24, N_hybrids_ = 27, *p* = 0.716; Figure 4) and in T_2_ (Mann–Whitney exact test: U = 624.00, N_pigs_ = 24, N_hybrids_ = 27, *p* = 0.114; Figure 4b).

**Figure 5:**
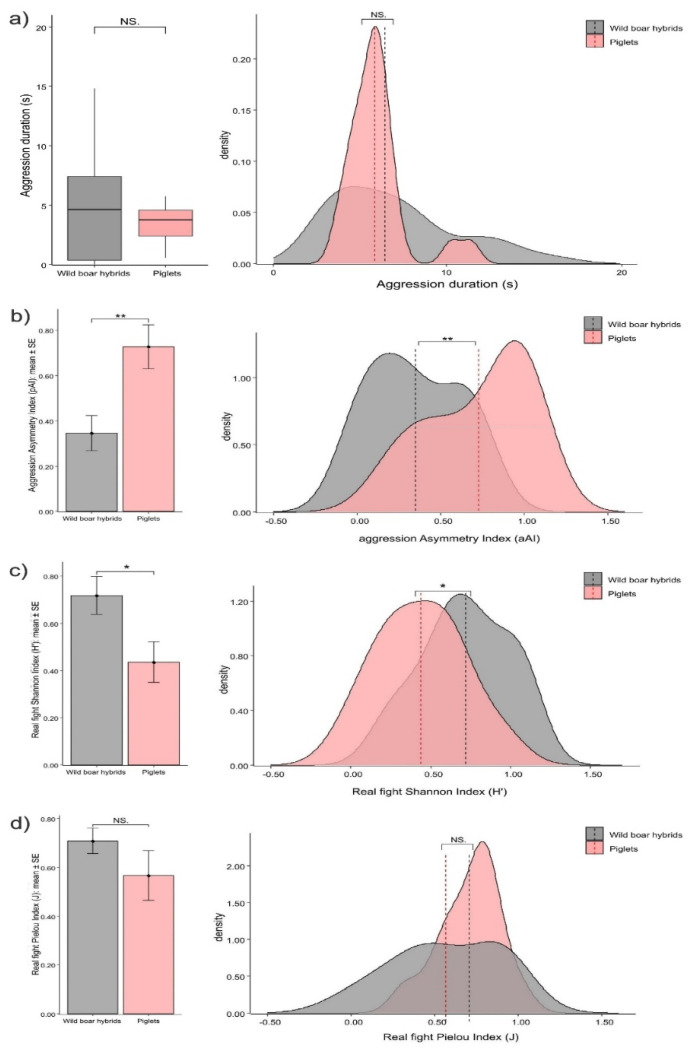
Differences in aggression duration between piglets and wild boar hybrids shown as bar plot (left) and density plot (right). (**a**) Piglets and wild boar hybrids show comparable levels in the aggression duration (Mann–Whitney exact test; *p* = 0.539) Density plot: vertical, dashed lines indicate median value. Bar plot: horizontal line: median value; box: interquartile range; vertical line: minimum and maximum values in the data. Difference in real fighting between piglets and wild boar hybrids shown as error bar plot (left) and density plot (right). (**b**) Asymmetry Index (aAI) values; piglets show top levels of asymmetry (*t*-test for independent samples; *p* = 0.007). (**c**) Shannon Index (H′) values; piglets show the lowest levels of real fight variability (*t*-test for independent samples; *p* = 0.027). (**d**) Pielou Index (J) values; piglets and wild boar hybrids show comparable levels of real fighting in the Pielou Index (evenness) (*t*-test for independent samples; *p* = 0.239). Density plot: vertical, dashed lines indicate mean value. Bar plot: vertical bars. Standard Error (SE) around the mean (circles). NS = non-significant, * = *p* < 0.05, ** = *p* < 0.01.

*Structure of aggression*. There was no significant difference in the aggression duration between piglets and wild boar hybrids (Mann–Whitney exact test: U = 591.00, N_pigs_ = 24, N_hybrids_ = 27, *p* = 0.533; Figure 5a) The asymmetry (aAI) of real fight sessions was higher in piglets than in wild boar hybrids (*t*-test for independent samples, N_pig_dyads_ = 10; N_hybrid_dyads_ = 12; t = −3.062; *p* = 0.007; Figure 5b). The level of pattern variability (H′) was significantly lower in piglets than in wild boar hybrids (*t*-test for independent samples, N_pig_dyads_ = 10; N_hybrid_dyads_ = 12; t = 2.384; *p* = 0.027; Figure 5c), whereas the levels of pattern evenness (J) was comparable between piglets and wild boar hybrids (*t*-test for independent samples, N_pig_dyads_ = 10; N_hybrid_dyads_ = 12; t = 1.232; *p* = 0.239; Figure 5d). 

## 4. Discussion

### 4.1. Social Play Levels and Structure in Piglets and Wild Boar Hybrids 

In this study we found that overall social play frequencies tended to be higher in piglets and wild boar hybrids (even though full significance was not reached; *Prediction 1a partly supported;* Figure 2a) and piglets engaged significantly more in social play than wild boar hybrids after the 20th day of life (T_2_ and T_3_; Figure 2b) (*Prediction 1c supported*). Clearly, considering wild boar hybrids and not 100% wild boars (with both wild boar parents) can have dampened the behavioural differences between the domestic and wild form. However, taken together, the above results indicate that the domestication syndrome expectations on social play levels were partly met. The fact that the frequency of social play is comparable in the first phase of development between piglets and wild boar hybrids (within the first 20 days of life, T_1_) is in line with the function of this behaviour. From the very beginning, social play is important to develop the physical and social skills that are necessary to enhance self and social assessment, conflict resolution, individual recognition, group cohesion, and social bonds [41,44,46,48,63,79]. However, in the litters under study, after the first developmental stage, play became highest in the more domestic form. One aspect of the domestication syndrome is neoteny, a slowing down in the developmental rate, which operates throughout all ontogenetic phases [80,81]. Play—in particular, its persistence during individual development—is considered one of the behavioural traits highlighting the neotenic nature of a species [14,15,82,83]. Accordingly, our results can support the neotenous feature of the domestic pig compared to its wild counterpart, since play fighting decreased over time proportionally less in piglets than in wild boar hybrids (Figure 2b).

Increased play levels have been observed also in other domesticated species. For example, the behavioural profile of the domestic dog seems to be broadly mappable onto that of wolves, although this overlapping appears less complete when considering the propensity to play [84,85]. Even if both domestic dogs and grey wolves intensively play to establish and develop social ties, a playful propensity appears to be more intense in dogs [86,87,88]. In primates, bonobos—which are considered as a self-domesticated species compared to chimpanzees—show higher levels of social play than chimpanzees [82,89,90,91]). 

Contrary to our expectation (*Prediction 1b not supported*), social play sessions in piglets were shorter and, with respect to play fighting, less uniform (J) and variable (H′) than in wild boar hybrids (Figure 3a–c). Moreover, the level of asymmetry (pAI) of play fight sessions was comparable (Figure 3a) between piglets and wild boar hybrids. Overall, these results suggest that piglet players were not able to engage in more successful play sessions (as per [53]) than wild boar hybrid players. Indeed, successful players should be more capable of balancing their offensive and defensive patterns, thus avoiding escalation into real aggression and ultimately prolonging play [51,52,83,92]. The fact that this ‘balancing ability’ is not so pronounced in the more domestic form may be related to the strong competitive nature of play fight in piglets, which can be used as a substitute for aggression and may possess some features of real fighting (e.g., rank determination [41]). In *Canis lupus*, both domestic dogs and wolves can show asymmetrical play fighting behaviours because, for example, subordinates escalate play fights to try to unseat dominant animals or to probe another’s physical abilities [93]. In this case, the competitive nature of play fight might also play a role in determining structural similarities between the wild and the domestic form. 

To our knowledge, the present literature does not allow the further elaboration on this aspect due to the lack of studies that directly compare domestic and wild forms with respect to social play structure.

### 4.2. Aggression Level and Structure in Piglets vs. Wild Boar Hybrids

Contrary to what we expected, the frequency of aggression was higher in piglets than in wild boar hybrids (*Prediction 2a not confirmed*; Figure 4a). The temporal analysis revealed that aggression levels were comparable in the first 35 days of life (T_1_ and T_2_) but became higher in piglets than in wild boar hybrids after the fifth week of life (T_3_) *(Prediction 2c not confirmed*; Figure 4b). These results apparently contradict what is expected from the domestication syndrome, according to which aggression levels should be lower in the domestic forms and lead to increased docility [55,56,57]. However, at a closer look, this may not be a correct interpretation. The traits of the domestication syndrome (increased docility and decreased aggressiveness) probably apply more to the relationship between animals and humans than to the relationship between conspecifics. This is in line with the definition of domestication provided by Pride and Hongo [27], who considers the traits of domesticated animals as the adaptation to the selection pressures of living within anthropogenic niches defined by management. Accordingly, studies on dogs have shown that domestication has been able to influence their social behaviour towards humans, with whom they establish preferential social bonds [93]. Dogs show lower levels of aggression and higher levels of avoidance towards humans than wolves in the same conditions [94], and a higher tendency to seek human social contact [95]. Thus, by reducing fear [96] and increasing docility to humans [97,98], domestication has shaped specific behavioural traits that favour the formation of stable bonds between domestic animals and humans. In addition, dogs can show avoidance towards conspecifics in order to avoid conflicts, rather than behaviours of reconciliation following aggression [99] as opposed to wolves, in whom frequent reconciliation behaviour has been observed [100,101]. Consistently, Hansen Wheat et al. [19] showed deviations between ancient and modern dog breeds for (among others) persistent aggressiveness, and they suggested that behavioural correlations related to domestication can be decoupled, leading to the maintenance of certain behavioural traits and the loss of others. Similarly, in *Sus scrofa*, different factors could have influenced aspects of domestication by shaping behavioural traits that vary depending on whether the interaction is with humans or conspecifics. The higher levels of aggression in the domestic pig may be the result of an increased competitiveness developed over the course of the domestication process as a possible by-product of the long-term cohabitation in rearing and confined spaces with patchy resources and no possible avoidance [31,46,54]. On the other hand, wild boars form groups that can frequently split into different subgroups, varying temporally in composition in relation to demographic and ecological factors. This situation may have resulted in a less stable and cohesive society compared to that of the domestic pig [31,63,101,102]. 

Similar constraints (forced cohabitation for a long time, space, and resources) may also explain why—compared to wild boar hybrids—piglets showed comparable aggression duration (Figure 5a) and more asymmetrical (aAI; Figure 5b) and predictable (less variable as per H′; Figure 5c) real fight sessions (*Prediction 2b not supported*). Asymmetrical agonistic interactions are crucial to acquire a dominant status within social groups, as the hierarchical rank of an individual increases as the result of repeated aggressive encounters that are consistently won by that individual [103,104,105,106,107] The dominance status deriving from such directional aggressive encounters allows high ranking individuals to access resources with priority, thus increasing their fitness [108,109,110,111,112,113]. As it occurs for social play, it is not possible to go deeper in the discussion of the differences in the structure of agonistic interactions due to the lack of studies that directly compare domestic and wild forms in this respect.

A main limit of this study is that we could not compare the domestic pigs with ‘full’ wild boar subjects, which may have dampened some differences between the domestic and wild form. A comparison between the domestic and wild form with a larger number of individuals and more than three litters, within a single season and with theextension of data collection after weaning could help gain more information about the behavioural differences that may have emerged as a result of domestication. In particular, data on various groups, also including different breeds and extensive farming conditions, would allow further inferences on domestication syndrome at the population level and allow the distinction between traits that have been preserved by thedomestication processes and more ‘volatile’ traits that are contingent on farming strategies. Some of these limitations are compensated for by the quite unique opportunity to collect video data in adjacent months on wild boar hybrids (whose birth was unplanned) and piglets that were (i) raise;d under the same (non-intensive) environmental setting; (ii) exposed to the same management conditions (including the type and timing of food provisioning); (iii) characterized by the same level of kinship across either wild boar hybrid and piglet litters and (iv) probably minimally affected by experience given their very young age. Moreover, our results were obtained by an in-depth behavioural video analysis on an extensive session sample (more than 600 sessions involving 51 individuals). 

## 5. Conclusions

In conclusion, our study sheds light on an interesting anthropological question, which is how artificial selection operated by *Homo sapiens* since Neolithic might have operated on behaviour to favour the transition from the wild to the domestic forms, namely *Sus scrofa*. Our results show that certain domestication traits are already present in the early stages of life but also that the domestication syndrome does not fully apply to either the playful or the aggressive domain. This mismatch is probably apparent and not actual, in that artificial selection in domestic pigs—as well as in other species—has probably produced greater tameness towards humans rather than towards conspecifics. This aspect has not been covered by this study and deserves further investigation in the future. 

## Figures and Tables

**Table 1:** Composition of the litters of piglets and wild boar hybrids.

Category	Litter	Individuals	Date of Birth
**Piglets**	1	11 (5 female; 6 male)	16 Sept 2018
	2	6 (2 female; 4 male)	3 Oct 2018
	3	8 (4 female; 4 male)	5 Nov 2018
**Wild boar hybrids**	1	9 (5 female; 4 male)	14 Jun 2018
	2	8 (5 female; 3 male)	05 Jun 2018
	3	10 (3 female; 7 male)	10 Jun 2018

**Table 2:** Ethogram with play patterns recorded in the current study.

Play Patterns			
Category	Behavioural Pattern	Type	Description
**Offensive**	Attempt play bite	C	A piglet attempts to bite the partner, but there is no contact with it
	Head play knocking	C	A piglet hits another individual with the head
	Play bite	C	A piglets bites a partner by delicately closing mouth over the other’s flesh
	Play lifting	C	A piglet attempts to displace a partner by lifting or levering it with snout or head
	Play mount/climb	C	A piglet places both front hoofs on the back of another piglet or sow
	Play push	C	A piglet drives its head, neck, or shoulders with minimal or moderate force into another piglet’s body. Occasionally, this pattern results in the displacement of the target animal. It is significantly more intensive than nudging
	Play run	LA	A piglet runs and hops in forward motions within the pen environment. Run can be performed both in solitary and social manner
**Neutral**	Flopping	LA	A piglet drops to the pen floor from a normal upright position to a sitting or lying position. There is no contact with an object or another individual that could cause the change in position
	Head tossing	LA	A piglet gently head shakes from one side to another
	Hopping	LA	A piglet has either its two front feet or all four feet off the pen floor at one time through an energetic upwards jumping movement. The piglet continues facing the same original direction for the whole of the behaviour
	Leg spreading	LA	A piglet spreads its fore and hind limbs and it moves quickly from side to side
	Nudge	C	A piglet uses its snout to gently touch another piglet’s body (excluding nose–nose contact). It is more intensive than touching, but also more gentle than pushing
	Object play	LA	A piglet manipulates an item or securely holds it in its mouth, energetically shaking it or carrying it around the pen
	Pivot	LA	A piglet twirls its body on the horizontal plane by a minimum of 90°. Pivot is usually associated with jumping on the spot
	Scamper	LA	A piglet performs two or more forward directed hops in quick succession of each other usually associated with excitability
**Defensive**	Play kneeling	LA	A piglet goes down on its knees while playing
	Play lying down	LA	A piglet places itself in a horizontal position during play
	Play sitting	LA	A piglet sits during play

Integrated or modified from other ethograms [44,46,47,62,63,64,65]. LA = locomotor/acrobatic pattern, C = contact pattern.

**Table 3:** Ethogram with aggressive patterns recorded in the current study.

Aggressive Patterns			
Category	Behavioural Pattern	Type	Description
**Offensive**	Aggressive bite	C	A piglet opens its mouth and closes its teeth tight on a small piece of the opponent’s flesh (except tail)
	Aggressive head knocking	C	A piglet lunges or jerks its head with physical contact and mouth closed
	Aggressive kick	C	A piglet kicks with one or both hind limbs the opponent, striking it
	Aggressive lifting	C	A piglet attempts to displace the opponent by lifting or levering it with snout or head
	Aggressive mount/climb	C	A piglet forces the opponent to move away by rising upon the rear of the partner
	Aggressive push	C	A piglet presses its head, neck, shoulder or body against the opponent in an aggressive context
	Attempt aggressive bite	C	A piglet opens its mouth, directs or turns its head towards the body of the opponent and closes its mouth without contact
**Neutral**	Head tilting	LA	A piglet moves the head to the side when the opponent passes or gets closer
	Threat	LA	A piglet arches the back to the opponent or makes a forward movement of the head and stares at the opponent with no physical contact
	Rest during fight	LA	A piglet rests and does not exhibit aggressive behavior while being hit-during a reciprocal real fight session for at least 3 s. (reciprocal fighting must occur before and after this event for it to be classified as a rest during fight).
**Defensive**	Asymmetric parallel	C	The piglets involved in a real fight face the same direction, standing side by side and one of them is slightly ahead of the other. A piglet—the one placed slightly in front of the other—moves forward, pushing the opponent away with his shoulder and moving his head away from the opponent to avoid having its ears bitten.
	Avoidance	LA	A piglet moves away with a depressed tail when the opponent approaches
	Flee	LA	A piglet runs away from the opponent. The opponent can react with a chase.
	Withdrawal	LA	A piglet tries to leave a reciprocal real fight session, the opponent continues to bite the recipient with a rate greater than one bite for 3 s, and the recipient reacts with any harmful aggression for more than 3 s. After that, the piglets involved do not have interactions for at least 3 s.

Integrated or modified from other ethograms [44,46,47,62,63,64,65]. LA = locomotor/acrobatic pattern, C = contact pattern.

## Data Availability

The data presented in this study are available as supporting material to this study [Data_S1.xlsx].

## Supplementary Materials

The following supporting information can be downloaded at: https://www.mdpi.com/article/10.3390/ani12182458/s1, Video S1: play fight in wild boar hybrids; Video S2: play fight in piglets; Video S3: real fight in hybrids; Video S4: real fight in piglets; Video_Legend S1: legends of supplementary videos (S1–S4).
