# Supplementary material for: Does the Domestication Syndrome Apply to the Domestic Pig? Not Completely
Source: Animals (Basel). 2022 Sep 17;12(18):2458. doi: 10.3390/ani12182458 (PMC9495052; doi:10.3390/ani12182458)
Supplement: Supplementary file 1 [file animals-12-02458-s001.zip › Supporting_material_list.pdf]

DOES THE DOMESTICATION SYNDROME APPLY TO THE DOMESTIC PIG? NOT COMPLETELY

Edoardo Collarini[1], Marika Gioia[1] , Giada Cordoni[1.a] and Ivan Norscia[1,a]

List of supporting material

- Data calculation sheet;
- Video examples showing piglets and hybrids during play fight and real fight (video legends below)

Legends of supplementary videos (S1-S4)

Video_S1

Fragment of play fight sequence between two wild boar hybrids. A play kneeling (a play signal) is performed by the darker, striped individual.

Video_S2

Fragment of play fight sequence between piglets. Hopping (a play signal) is performed by the individual with only one black patch on the back.

Video_S3

Fragment of real fight sequence between wild boar hybrids. No play signals are present whereas attempt bites are present. One of the two individual flees.

Video_S4

Fragment of real fight sequence between piglets. No play signals are present whereas attempt bites are present. One of the two individual flees.
